# Pharmacy services for the 2019 Fédération Internationale de Natation (FINA) World Masters Championships in Gwangju, South Korea

**DOI:** 10.1186/s13102-021-00329-6

**Published:** 2021-08-23

**Authors:** In Kyu Yang, Eun Ok Shin, Dong Gyun Kim, Hyun Cheol Jung, Kwang Joon Kim, Sung Hwan Ki

**Affiliations:** 1 Gwangju Pharmaceutical Association, Gwangju, Republic of Korea; 2 College of Pharmacy, Mokpo National University, Muan-gun, Jeonnam 58554 Republic of Korea; 3 College of Pharmacy, Chosun University, Pilmun-daero 309, Dong-gu, Gwangju, 61452 Republic of Korea

**Keywords:** Pharmacy services, FINA, Gwangju, Athletes’ village pharmacy

## Abstract

**Background:**

The role of sports pharmacists is being emphasized in international athletic events. This study aimed to describe the pharmacy services for the 2019 Fédération Internationale de Natation (FINA) World Masters Championships in Gwangju, South Korea.

**Method:**

Research focused on athletes and coaching staff who received medications after visiting medical centers and pharmacies located in the athletes’ village from July 5 to July 29, 2019. We collected daily results of pharmacy operation and prescription interventions. The data were analyzed using Microsoft Excel, and were expressed as frequency (%).

**Results:**

Throughout the tournament, 633 patients received medication at the athletes’ village pharmacy (gender: 338 men [53.4%], 295 women [46.6%]; nationality: 299 Korean [47.2%], 334 overseas players [52.8%]; patient type: 150 athletes [23.7%], 427 non-athletes [67.5%]). Therapy for musculoskeletal disorders was the most common (n = 29, 19.3%), and oral NSAIDs (n = 56, 22.0%) were the most frequently dispensed medication in athletes. Pharmacists intervened for 47 out of 491 prescriptions (9.6%), with dosage change (n = 21, 44.7%) being the most common intervention type.

**Conclusion:**

Sports pharmacists at FINA World Masters Championships played a pivotal role in ensuring the safe usage of medications by all participants, especially athletes. This study results will be a useful reference for pharmacy services at future international or domestic sports competitions.

**Supplementary Information:**

The online version contains supplementary material available at 10.1186/s13102-021-00329-6.

## Introduction

Sports pharmacy is an evolving specialty within pharmacy practice and the role of sports pharmacists in sports medicine has been emphasized. Over the past decades, some sports pharmacy operations have been reported in major international athletic competitions [1–4]. Sports pharmacists are responsible for providing drug education, information services, and counseling on anti-doping to athletes, coaches, and the public, as well as injury management and prevention, and first aid at all levels of competition.

As most athletes seek substances or techniques to improve their performance, some try to use inappropriate methods, such as drugs or supplement abuse and taking anabolic steroids. Therefore, an expert sports pharmacist especially requires knowledge of the World Anti-Doping Agency [5] banned drugs and supplements for providing appropriate pharmacotherapeutic recommendations. Following the International Pharmaceutical Federation (FIP) Guidelines in 2014, three recommendations for sports pharmacists are included: (1) “keeping up-to-date with the contents of the WADA code,” (2) “assisting athletes to recognize whether the use of a substance may be banned or restricted in their sport”, and (3) “providing information to athletes about the risks and benefits of nutritional supplements” [6, 7].

FINA World Masters Championships, an international aquatics championship for adults, is organized biennially by the Fédération Internationale de Natation (FINA). The 18th FINA World Masters Championships was held in Gwangju metropolitan city of South Korea in 2019. For 17 days from July 12 to July 28, 7456 athletes from 91 countries competed in six sports events in five stadiums. During the competition, a pharmacy in the athletes’ village medical center was established. The purpose of the pharmacy was to ensure that athletes who required medical services during the competition were prevented from accessing drugs prohibited by WADA. In addition, prescription audits and information services were provided to prevent inappropriate drug use.

This might be the first time a pharmacy was operated by a local pharmaceutical association in an international sports event, and also the first time a pharmacy service was offered at the FINA World Masters Championships. The purpose of this study is to review and share the performance and experience of pharmacy operations at the 2019 FINA World Masters Championships.

## Methods

This study was conducted retrospectively and all data were reported using descriptive statistics. All experimental protocols were approved by the Gwangju organizing committee of FINA World Masters Championships.

### Study subject and period

This study focused on athletes and coaching staff who received medication after visiting the medical centers and pharmacies located in the athletes’ village from July 5 to July 29, 2019.

### Data collection and analysis

The data for this study included the training and conference data organized by the Gwangju Pharmaceutical Association in order to establish and operate the pharmacy. For the baseline characteristics of the study group, we collected information on gender, nationality, patient type, diagnosis and medication names. In addition, we collected daily results of pharmacy operation and prescription interventions; total number of prescriptions, type of medication, total number of pharmacists' interventions and type of intervention. The collected data were analyzed using Microsoft Excel and results were expressed as counts and percentages.

## Results

### Recruitment of pharmacists and pre-education courses

The Gwangju Pharmaceutical Association, which was the main operator of pharmacy service for the 2019 FINA World Masters Championships, had employed three full-time pharmacists to provide pharmacy service. In addition, 33 volunteer pharmacists were recruited from all over Korea.

Pre-education courses for full-time and volunteer pharmacists were provided three times through on-site lectures. The first training was based on a case study of a former sports pharmacy operated at the 2018 Pyeongchang Winter Olympics in South Korea. The second training focused on the pharmacy operation manual, the list of medications to be used, and the prohibited medications. Lastly, pharmacy tours were conducted by individuals. After completion of the training, the effectiveness of training for participating pharmacists was checked through Q&A and discussion time.

### Pharmacy handbook publication and medication list

In addition to prior training for full-time and volunteer pharmacists, a pharmacy handbook was prepared and provided to them. The pharmacy handbook contained basic information on the operation of the pharmacy, a list of medications, and WADA approval for each medication (Additional file 1).

The pharmacy medication list was prepared in Korean and English, with a total of 21 therapeutic categories and 113 medications. The medication list provided information on therapeutic class, ingredient name, brand name, dosage form, contraindications, and storage method, and additionally described the usage, route of administration, side effects, and WADA acceptance information for each medication (see Table 1). Narcotics were not included in the medication list.

**Table 1:** Analysis of medication list

Therapeutic category	n (%)
**Total**	**113 (100)**
Cardiovascular	15 (13.3)
Gastro-intestinal system	12 (10.6)
Antimicrobial agents	12 (10.6)
Analgesics and anti-inflammatory	10 (8.8)
Ophthalmic	9 (8.0)
Dermatological	9 (8.0)
Respiratory system	8 (7.1)
Fluids	6 (5.3)
Autonomic nervous system	4 (3.5)
Disinfectants	4 (3.5)
Endocrine system	3 (2.7)
Hormones and antagonistic	3 (2.7)
Antihistamines	3 (2.7)
Otorhinolaryngological	3 (2.7)
Local anesthetics	3 (2.7)
Skeletal muscle relaxants	2 (1.8)
Dental	2 (1.8)
Biological	2 (1.8)
Central nervous system	1 (0.9)
Vitamins	1 (0.9)
Emergency contraceptive	1 (0.9)

### Pharmacy operation and dispensing system

A pharmacy was set up in the athletes' village medical center from July 5 to July 29. The pharmacy operated from 9 am to 9 pm. One full-time pharmacist and one volunteer pharmacist worked in the pharmacy in 6 h shifts (9 am–3 pm and 3 pm–9 pm).

The role of the pharmacy consisted of prescription audit, preparation of prescriptions, medication guidance, safe storage and management of medicines, medication use management, adverse drug reaction management, and evaluation of drug use in athletes' village. In particular, pharmacists had to confirm the identity of the patient through open-ended questions during the prescription audit stage. If the patient was an athlete, the pharmacist checked whether the prescribed medication corresponded to a prohibited substance. When dispensing a medication approved by the Therapeutic Use Exemption (TUE) Committee, the physician who issued prescription was asked to confirm once again, and a prohibited medication stamp was placed on the prescription prior to dispensing. Medication was limited to a three day supply and injections were dispensed directly to nurses for administration. An electronic software program, PharmIT3000, supported by Korea Pharmaceutical Information Center, was used for medication dispensing.

### Characteristics of study group

A total of 633 patients, including athletes and coaching staff, received medication at the athletes’ village pharmacy during the competition. Among these patients, 338 were men (53.4%) and 295 were women (46.6%), 299 were Koreans (47.2%) and 334 were overseas players (52.8%). Majority of the prescriptions related to internal medicine (n = 368, 58.1%), sports medicine (n = 128, 20.2%), ophthalmology (n = 63, 9.9%), and emergency medicine (n = 61, 9.6%). Patients who needed internal medicine care or orthopedic treatment visited internal medicine and sports medicine, respectively. However, patients who needed first aid such as acute muscle pain or diarrhea visited emergency medicine. Athletes accounted for 150 (23.7%) of the total patients, with majority of them being musculoskeletal therapy patients (n = 29, 19.3%) (see Table 2).

**Table 2:** Characteristics of study group

Category	n (%)
**Total patients who visited the pharmacy**	**633 (100)**
**Gender**	
Male	338 (53.4)
Female	295 (46.6)
**Countries**	
South Korea	299 (47.2)
Overseas players	334 (52.8)
**Patient type**	
Athletes	150 (23.7)
Non-athletes	427 (67.5)
Unknown	56 (8.8)
**Number of sports events**	**6**
**Medical department**	
Internal medicine	368 (58.1)
Sports medicine	128 (20.2)
Eye clinic	63 (9.9)
Emergency room	61 (9.6)
Dental clinic	7 (1.1)
Unknown	6 (0.9)
**Diagnosis of athletes**	**150 (100)**
Musculoskeletal	29 (19.3)
Gastrointestinal	16 (10.7)
Dermatology	15 (10.0)
Ophthalmology	12 (8.0)
Respiratory	9 (6.0)
Ear, nose, throat	9 (6.0)
Urology	4 (2.7)
Neurology	4 (2.7)
Dental	1 (0.7)
Unknown	51 (34.0)

**Fig. 1:**
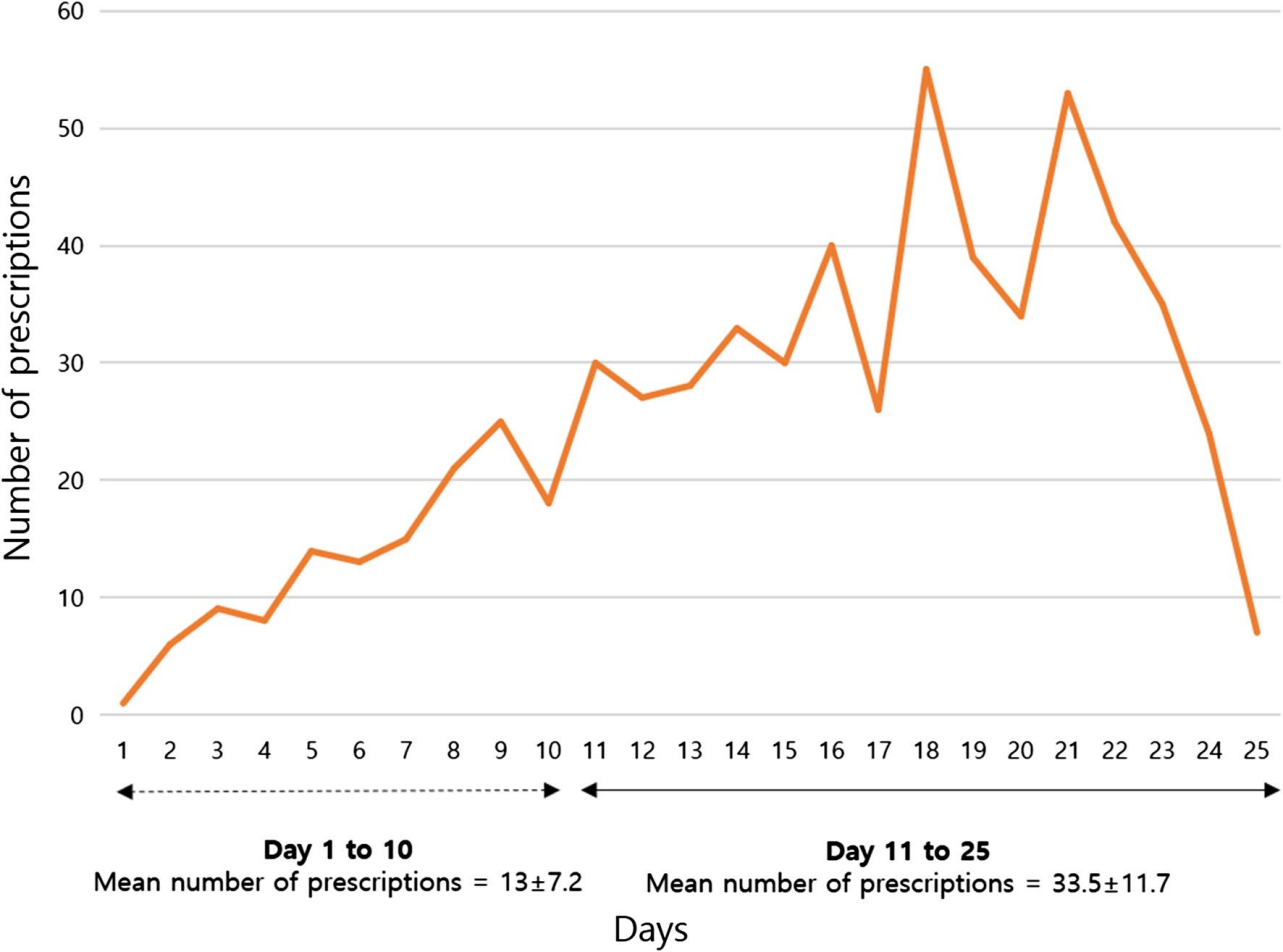
Number of daily prescriptions during the FINA World Masters Championships Gwangju 2019

The average number of prescriptions issued daily was 25.3 (± 14.3). There were less than 13 (± 7.2) prescriptions dispensed daily over the first 10 days of the competition, while the number rose to 33.5 (± 11.7) for days 11–25 of the competition (see Fig. 1).

As for the route of administration to athletes, oral medication accounted for the largest portion (194 cases, 76.4%), followed by external preparations (49 cases, 19.3%) and injections (11 cases, 4.3%). Furthermore, majority of the prescriptions comprised analgesic and anti-inflammatory drugs, followed by gastrointestinal drugs, respiratory drugs, and antimicrobials (see Table 3).

**Table 3:** Analysis of prescriptions issued to athletes

Category	n (%)
**Total number of athletes**	**150 (100)**
**Total number of prescription medicines of athletes**	**254 (100)**
**Route of administration**	
Oral (P.O.)	194 (76.4)
Topical and local*	49 (19.3)
Injection†	11 (4.3)
**Analgesics and anti-inflammatory agents**	
NSAIDs	56 (22.0)
Acetaminophen	21 (8.3)
**Gastrointestinal agents**	
Antiulcer	29 (11.4)
Digestive	6 (2.4)
Antidiarrheal	4 (1.6)
**Respiratory agents**	
Antihistamines	23 (9.0)
Antitussives	23 (9.0)
Inhalants	1 (0.4)
Antimicrobials	21 (8.2)
Muscle relaxants	9 (3.5)
Others*^†‡^	**62 (24.4)**

*Topical and local; topical NSAIDs (9), topical steroids (2), inhalants (1), antimicrobial eye drops (9), artificial tear (8), steroid eye drops (5), antihistamine eye drops (2), antimicrobial ear drops (7), antimicrobial ointment (6)

^†^Injection; diclofenac (5), tramadol (1), ketorolac (1), ambroxol (1), bropium (1), metoclopramide (1), vaccine (1)

^‡^Emergency contraceptives (2)

### Prescription intervention by pharmacists

Prescription interventions by pharmacists were recorded for 18 of the 25 days during the competition. The results showed interventions for 47 (9.6%) of the 491 prescriptions. As for the types of interventions, change in dosage (21 cases, 44.7%) accounted for the majority of interventions, followed by change in number of prescription days (9 cases, 19.1%) and dispensing after confirming the prescription from doctor (9 cases, 19.1%). WADA-related prescription interventions happened in 3 (0.6%) cases; 1 case related to normal saline 500 mL containing a multi-vitamin, 1 case related to a beta-2 agonist inhaler, and 1 case related to oral iron medicine, respectively (see Table 4).

**Table 4:** Analysis of prescription intervention by pharmacists (18 days)

Category	n (%)
**Total prescriptions**	**491 (100)**
Total interventions†	47 (9.6)
Total interventions^†^ in respect of WADA^‡^	3 (0.6)
**Type of medicine**	**47 (100)**
Oral	34 (72.3)
External for skin	8 (17.0)
Injection	4 (8.5)
Ophthalmic	1 (2.1)
**Type of intervention**	**47 (100)**
Change of medicine dosage	21 (44.7)
Change of prescription days	9 (19.1)
Dispensed after confirmation of prescription	9 (19.1)
Change of medicine	3 (6.4)
Change of medicine usage	2 (4.6)
Medicine added	2 (4.6)
Report of adverse drug reaction	1 (2.1)

^**†**^Total duration of the FINA competition was 25 days, of which the daily record of prescription intervention had been completed only for 18 days. Total number of prescriptions for 18 days was 491

^**‡**^WADA; World Anti-Doping Agency; 1 case related to 1 case related to normal saline 500 mL containing a multi-vitamin, 1 case related to beta-2 agonist inhaler, and 1 case related to oral iron medicine

## Discussion

The need for safe use and efficient management of athletes' village pharmacies continues to grow in international sports competitions. In this paper, we reviewed the performance of the athletes' village pharmacy operated at the 2019 FINA World Masters Championships, Gwangju, South Korea.

Systematic preparations to manage a pharmacy at the athletes' village medical center were launched four months before the start of the competition. The pharmacy in the athletes' village was run by the local pharmaceutical association. The Gwangju Pharmaceutical Association first formed a preparatory team to establish a plan to operate a pharmacy in the athletes' village and recruit full-time and volunteer pharmacists. Recruited pharmacists focused their training on sports pharmacy related to WADA, International Olympic Committee (IOC) Needle Policy, and TUE through three training programs. During the operation period of the pharmacy, information related to medication was periodically collected and assessed. In particular, the full-time pharmacist reviewed daily prescriptions for details of medication use, and looked after the health of athletes and coaching staff in the athletes' village. They reported to officials of the Gwangju organizing committee of the FINA World Masters Championships if the use of certain drugs, such as anti-diarrhea and antibiotics, increases. According to past studies, infectious diseases had a significant effect on athletes’ performance [8, 9]. Therefore, pharmacists intensively monitored the occurrence of infectious diseases in athletes’ village, including the rate of prescriptions for anti-diarrheals and antibiotics.

Pharmacists play a major role in doping control programs, and can prevent athletes from inadvertently consuming banned substances [10]. In addition, pharmacists conducted prescription audit, medication guidance, and evaluation of drug use in FINA World Masters Championships. As a results of pharmacy operation and pharmacists’ interventions, a total of 633 patients visited the pharmacy during the competition, of which 150 were athletes. In particular, 9.6% of prescription interventions were carried out in the pharmacist preparation process and there were three WADA-related interventions. Though compared to the Summer or Winter Olympics, the total number of participants was relatively small at the FINA World Masters Championships, the most frequently administered medications to athletes were analgesics and anti-inflammatory agents similar to those at the Pyeongchang Winter Olympics or the London Olympics [1, 2]. This is the first study report on the frequency and case of a prescription intervention at an international sports competition, and systematic prescription intervention by pharmacist is particularly important in sports medicine, which consists of various medical teams.

The FINA athletes' village pharmacy operating system was improved after review of other successful sports pharmacy operations. A separate cabinet for WADA-banned medications was prepared, and when such medications were prescribed, pharmacists were required to ask the medical team to prevent incorrect administration. Meanwhile, during the London Olympics when a WADA-banned medication is prescribed, a warning appears and a pharmacist enters their password in the prescription system to confirm whether the patients are aware of the drug's status.

Although international sports competition have emphasized the appropriate use of medication by participants, there are insufficient guidelines for applying the same to many international sports events, such as the Olympics or the World Swimming Championships. FINA athletes' village pharmacy thus paid attention to successful previous case of sports pharmacies operating at the Pyeongchang Winter Olympics During the pre-education course, participant pharmacists became aware of the importance of the needle policy that the FINA organizing committee had overlooked and shared related information with the committee. As a result, when an athlete was prescribed normal saline 500 mL containing a multi-vitamin, a pharmacist intervention ensured that the needle policy was not violated. Therefore, it is important to review the previous operational cases and apply them to the present situation to establish a systematic and secure medication system in preparing for the athletes' village pharmacy.

## Conclusion

In current study, the activities and achievement of a pharmacy were recorded during the 2019 FINA World Masters Championships, Gwangju, South Korea. To the best of our knowledge, this is the first such study on the operation and performance of pharmacies at the FINA World Masters Championships. Therefore, this report might be a useful reference for pharmacy operations at large international or domestic sports events in the future.

### What are the findings?

- A pharmacist plays a pivotal role in ensuring the safe usage of medications by all participants, especially athletes.
- For effective pharmacy service, a pre-training course related to sports pharmacy operation examples in past international competitions is essential.
- Prescription intervention by pharmacist is crucial in sports medicine, which consists of various medical teams, to provide optimal health care for athletes.
- Since accumulated data for sports pharmacies is still lacking, it is very important to record the activities and performance of the sports pharmacy during the competition to inform future competitions.

### What is the limitation of the current study?

- Elaborate & objective evaluation of the effectiveness of the training was not conducted during or after the completion of the training course.
- Investigation on non-prescription drugs or supplements usage by professional swimmers has not been conducted.

### How it impacts clinical practice in the future?

- Our successful experiences of pharmacy services at major international swimming competitions might be useful for planning future sports pharmacy operations at major sports events.

## Supplementary Information

**Additional file 1**. The pharmacy handbook.

## Data Availability

The dataset used in the current study is available from the corresponding author on request.
