# Supplementary material for: Pharmacy services for the 2019 Fédération Internationale de Natation (FINA) World Masters Championships in Gwangju, South Korea
Source: BMC Sports Sci Med Rehabil. 2021 Aug 23;13:98. doi: 10.1186/s13102-021-00329-6 (PMC8383403; doi:10.1186/s13102-021-00329-6)
Supplement: Supplementary file 1 — Additional file 1. The pharmacy handbook. [file 13102_2021_329_MOESM1_ESM.pdf]

Pharmacy Services for the 2019 Fédération Internationale de Natation (FINA) World Masters Championships in Gwangju, South Korea

In Kyu Yang[1], Eun Ok Shin[1], Dong Gyun Kim[1] , Hyun Cheol Jung[1] , Kwang Joon Kim[2,‡], Sung Hwan Ki[3,†]

[1]Gwangju Pharmaceutical Association, Gwangju, Republic of Korea; [2]College of Pharmacy, Mokpo National University, Jeonnam, Republic of Korea; [3]College of Pharmacy, Chosun University, Gwangju, Republic of Korea

'Supplementary Data'

Pharmacy guide for 2019 FINA World Championships in Gwangju

1. Introduction

This guide contains information on the operation of the Pharmacy at the 2019 FINA World Championships in Gwangju and the medications provided, especially those prohibited from the World Anti-Doping Agency (WADA).

The publishers of this Guide have done their best to ensure that the information is accurate and up-to-date based on reliable data. Physicians should refer to the information and treat patients based on their expert knowledge.

The medications listed in this Guide are annotated with their status according to the 2019 WADA Prohibited List. We recommend that all athletes and medical staff familiarize themselves with it. The 2018 WADA Prohibited List is available at http://www.fina.org/content/prohibited-list.

In case a certain medication is required, but is not in this Guide, we will provide full assistance in acquiring it.

Welcome to the 2019 Gwangju World Swimming Championships in Gwangju.

I wish you the best of luck in your endeavors.

Hyuncheol Jung, Head of Pharmacy Service, President of Gwangju Pharmaceutical Association

EunOk Sin, Deputy Head of Pharmacy Service, Female President of Gwangju Pharmaceutical Association

Advisors

Sandy Rhie, , Professor, College of Pharmacy, Ewha Womans University

Sung Hwan Ki, Professor, College of Pharmacy, Chosun University

2. Pharmacy information

For the successful operation of the 2019 FINA World Championships in Gwangju, we hereby confirm the operation of the Pharmacy in the Medical Center for safe pharmacotherapy under professional pharmaceutical training.

2-1 Location and Operating Hours

The Pharmacy is located at the Medical Center in the World Championships Village.

Operating Hours: 9:00~21:00

Operating Period: July 5th ~ 29th, August 5th ~ 18th of 2019.

Two pharmacists will work in two shifts.

2-2 Pharmacist's Duties

1) Prescription audit
2) Compounding and dispensing
3) Keeping control over the requisition and dispensing of all medicine
4) Medication instruction and administration
5) Proper dispensing and management of narcotics
6) Safe management of inventory
7) Managing use of all pharmaceuticals
8) Maintaining sufficient stock at the infirmary
9) Managing adverse drug reactions
10) Preparation of needed reports
11) Checking the quality of all pharmaceuticals in the Village

3. Dispensing and Administration

The pharmacist examines the regulations needed in proper pharmacotherapy for the betterment of patients' performance, and conducts proper audits in prescription and dispensaries, closely managing compounds and providing medication instructions with precision under a sterile environment.

3-1 Verifying Information

Upon receiving the prescription, verify the name of the patient, issuing physician, and prescribed medicine, along with the usage, dosage, period of intake; and check if the drug is a controlled substance.

3-2 Preparing Envelopes

The envelope must be different for transdermal and oral medications, and drugs for special use should be separated even within the same patient. i.e.) take as needed, at bedtime, etc

3-3 Prescription audit

1) Before compounding and dispensing, the pharmacist must confirm the patient's identification through open-ended questions.
2) If the patient is an athlete, screen the prescription for prohibited drugs according to the data; then mark the confirmed information (O or X).
3) Check with the prescribing physician before preparing the drugs approved by the TUEC. Prohibited drugs must be marked on the prescription before dispensing. Also, address the prescription with the patient.
   (The above process further applies to drugs not approved by the TUEC.)
4) A copy of the TUE application and TUEC decision shall be kept at the pharmacy in the Medical Center.
5) If there are any anomalies in poly-drug use, dosage, or usage upon audit, the pharmacist must inquire about additional information from the prescribing physician.

3-4 Administration

1) Before administering, the pharmacist must confirm the patient's identification through open-ended questions, and offer appropriate medication guidelines to provide necessary

knowledge on the correct use of the medication. The guidelines will include the effect, usage, dosage, precautions and proper storage of medicine. If necessary, the pharmacist will provide the patient with guidelines, using online information or medication brochures.

2) The pharmacist only administers oral and transdermal medications. Intradermal injections shall be dispensed by the nurse.

3) Compounded medications can be administered up to 3 days, and injection should only be administered each session.

4) Upon administration, medication instructions should be provided(if necessary).

5) Only single-use administration is offered at the infirmary.

4. Prescribing a Prohibited Substance

Prohibited substances according to the 2019 WADA Prohibited List may not be used by athletes, unless a Therapeutic Use Exemption (TUE) has been granted, or the medication is administered in the course of emergency medical treatment.

Prescribing prohibited substances for athletes should be avoided, unless a TUE is obtained in advance. In medical emergencies an athlete's health takes absolute priority and treatment must not be delayed. A Retroactive TUE should be obtained as soon as the emergency treatment has been given (for example, opiates for severe acute pain relief).

If a TUE has already been obtained, a copy of the TUE documentation should be presented to the dispensing pharmacist, who will then dispense the medicine for the athlete.

5. Needle policy

Intravenous injection of 50 ml or more within 6 hours is prohibited (except for cases of emergency, surgery and clinical examination).

When administered, it must be recorded.

6 .Narcotic Control Guideline

we comply with the Korean Guideline on Narcotic Control for the medical use of narcotics. The Korean Guideline on Narcotic Control stipulates necessary measures for the effective management of narcotics, and prohibits the abuse of controlled substances.

.

6-1 Narcotics Handler Compliance

1) One cannot handle Narcotics unless he/she is a Narcotics Handler, and Narcotics can only be handled within the scope of work.
2) One cannot administer and/or dispense Narcotics, unless they are purchased from a Medical Center for administration.
3) One cannot purchase or transfer Narcotics from unqualified people who are not a Narcotics Handler.
4) A Narcotics Handler cannot transfer Narcotics other than those confirmed by relevant regulations.

6-2 Handling of Narcotics

1) Upon administering Narcotics, one must record the name of illness, the name of the Narcotics, the dosage, and the number of injections on the medical record. After confirming that the Narcotics are administered, the prescription should be printed out.
2) In Narcotics prescriptions, one must enter the address of the business, name of the business, name of the issuing physician with his/her license number in capital letters, and then sign or seal it. In addition, the patient's address, name, sex, age, the name of illness, and the date of issue of the prescription should be recorded.
3) When making a correction to the Narcotics prescription, draw two lines where it needs to be corrected, and then have the prescribing physician sign or seal it.
4) Narcotics injections can be administered 1 dose per prescription; if needed, the dose may be increased within the maximum daily dose.
5) If less than 1 ampoule was injected, the remaining Unused Narcotics should be returned to the Pharmacy immediately. Also, upon fluid mix, the mixture and used syringe must be returned as it was used. The Returned Unused Narcotics Record must be filled in and signed.
6) If the Narcotics were dispensed but not administered to a patient, only return the reusable Narcotics.

7. Pharmaceutical management guideline

The Pharmaceutical Department will manage the inventory (3 days standard), expiration dates, and special medication for the safe, appropriate, and thorough administration of pharmaceuticals.

7-1 Storage of pharmaceuticals outside the Pharmacy

1) Pharmaceuticals outside the Pharmacy can only be stored at the infirmaries within the Village.

2) Pharmaceutical storage at the infirmaries must follow the storage guideline of the Pharmacy.

3) Narcotics stored/managed at the infirmaries must follow the"Narcotic Control Guideline"

8. Reports and evaluation

If there are any incidents of adverse reaction to a prescribed medication, one must file a report and notify the prescribing physician.

The pharmacist will prepare a daily report to evaluate pharmaceutical usage so that it can be used to prepare for pharmacy operation in the future. The report includes trends in pharmaceutical usage by country, by efficacy group, et cetera.

Adverse Reaction Report

1. PATIENT INFORMATION

NAME	REGISTRATION #	AGE	GENDER	DIAGNOSIS(CODE)
			M / F	

2. PRESUMED CAUSE

NAME OF MEDICATION (COMPONENT)	ADMINISTRATION PERIOD
	yyyymmdd ~ yyyymmdd

3. CLINICAL REPORT

ADVERSE REACTION INFORMATION (ADE/ADR)			
SYMPTOM DIAGNOSIS			
OCCURANCE (start)	OCCURANCE (end)		DURATION
ymd am/pm___h)	y md(am/pm_____h)		00:00
MOMENT OF ADE/ADR	FIRST REACTION OCCURREDAFTER(i.e.,30 sec, 5 min, 2 hrs, 3 days, etc.)		
RESULT	□FULLY RECOVERED□RECOVERING□NOT RECOVERED□RECOVERED WITH SEQUELAE□UNKNOWN		
TREATMENT SUSPENSION	□SUSPENDED□NOT SUSPENDED□UNKNOWN	RE-ADMINISTRATION	□PERFORMED□SUSPENDED□UNKNOWN
EPIDERMAL REACTION	□PSORIASIS: □WHOLE BODY□PARTS ()<br>□LESION: □WHOLE BODY□PARTS ()<br>*CONDITION□MACULA/PAPULARASH□URTICARIA(HIVES)□ANGIOEDEMA□VESICLE□PUSTULE□ACNE□PIGMENTATION□DEPIGMENTATION□ALOPECIA<br>□MUCOUS MEMBRANE (SPEC.:□OCULAR□LABIAL□ORAL□PROCTAL/ANAL□GENITAL) □OTHERS:<br>□SKIN EXFOLIATION (SPEC:□FACE□SCALP□EXTREMITIES□CHEST□BACK□STOMACH<br>□WHOLE BODY□OTHER:	CRITICAL REACTION	**IN CASE OF CRITICAL ADE/ADR** (CHECK ALL APPLICABLE)<br>□DEATH<br>□ADMITTED AND/OR EXTENDED ADMISSION<br>□CAUSED DEFORMITY<br>□CRITICALLY SEVERE STATE<br>□CRITICAL DISABILITY AND/OR DISFUCTION<br>□OTHER CLINICALLY CRITICAL STATE
RESPIRATORY	□NASAL DISCHARGE□STERNUTATION□NASAL CONGESTION<br>□DYSPNEA□TUSSIS/COUGH□SPUTUM	CARDIOVASCULAR	□TACHYCARDIA□ARRHYTHMIA□PECTORALGIA<br>□HYPOTENSION(/ mmHg) □SYNCOPE<br>□OTHERS( )

	□TRACHYPHONIA□OTHERS(　　　)		
NEUROLOGICAL	☐DECLINE IN CONSCIOUSNESS<br>☐HEADACHE☐HYPOMNESIA☐VIRTIGO<br>☐LETHARGIC☐APHASIA<br>☐TREMOR☐DYSKINESIA☐DYSBASIA☐NUMBNESS<br>☐SLEEP DISORDER	GASTROINTESTINAL	☐ NAUSEA ☐ VOMIT ☐ DYSPEPSIA ☐ ABDOMINAL PAIN<br>☐ GASTROINTESTINAL BLEEDING(UPPER/LOWER) ☐ DIARRHEA<br>☐ CONSTIPATION ☐OTHERS(　　)
PSYCHIATRIC	☐DEPRESSION ☐DELIRIUM☐ ANXIETY<br>☐HYPERSENSITIVITY ☐ HYPERACTIVITY<br>☐MENTAL DERANGEMENT	HEPATOBILIARY	☐ELEVATED LIVER ENZYMES ☐ JAUNDICE<br>☐OTHERS(　　　)
SYSTEMIC	☐ FEVER ☐ WEIGHT LOSS ☐ WEIGT GAIN<br>☐ LOSS OF APPETITE ☐ GENERALIZED EDEMA<br>☐ HYPOTHERMIA ☐ ARTHRALGIA ☐ MYALGIA<br>☐ LYMPHADENOPATHY ☐ MALAISE ☐ OTHERS(　)	RENAL	☐ HEMATURIA ☐ ALBUMINURIA ☐ DIABETES<br>☐ RENAL FAILURE<br>☐ OTHERS(　　　)
HEMATOLOGIC	☐LEUKOPENIA ☐ANEMIA ☐ THROMBOCYTOPENIA<br>☐NEUTROPENIA ☐EOSINOPHILIA<br>☐OTHERS(　　　)	OTHERS	☐ DYSURIA ☐ LOSS OF SEXUAL DESIRE ☐ SEXUAL DYSFUNCTION<br>☐ MENSTRUAL IRREGULARITY ☐ HEARING DISORDER ☐ TINNITUS<br>☐ CONJUNCTIVAL CONGESTION ☐ VISUAL IMPAIRMENT<br>☐ OTHERS(　　　)

4. REPORTING PERSONEL

DATE	INSTITUTION	QUALIFICATION	NAME
201 ymd		□PHYSICIAN·DENTIST·KOREANMEDICINE<br>□PHARMACIST· □NURSE<br>☐PATIENT ☐(　)	

(Narcotics) PRESCRIPTION

DEPARTMENT		DATE	
CLASSIFICATION OF PATIENT IDENTIFICATION NUMBER		PRESCRIBING PHYSICIAN	(인)

P A T I E N T IDENTIFICATION N U M B E R		L I S E N C E N U M B E R	
PATIENT NAME		PHARMACIST S I G N	
AGE / GENDER		RECEIPIENT	
A D D R E S S			
D I A G N O S I S (K C D CODE)			
PRESCRIPTION DETAIL			

NAME OF MEDICATION
UNIT
DOSAGE
EACH VOLUME
FREQUENCY
DURATION
METHOD
SCALE
MAX VOLUME
UNUSED VOLUME

2019 FINA World Championships Pharmacy Services

ATHLETE CONSENT TO RECEIVE WADA PROHIBITED SUBSTANCES

Date:

Name, strength and quantity of prescribed medication:

I confirm that I am aware that the above mentioned medication dispensed by the Pharmacy is Prohibited in sport according to the 2019 World Anti-Doping Agency Prohibited List.

I am aware that a Therapeutic Use Exemption (TUE) is required to be applied for and approved by the FINA TUE Committee before taking the medication.

I am aware that failure to have an approved TUE for the medication above may result in an Adverse Analytical Finding, and may be subject to disciplinary action.

I confirm that a TUE has been approved for the medication and is currently valid.

Athlete Name

Athlete Accreditation

Athlete Signature

Country

____

Witnessed by:

Dispensing Pharmacist Name

Dispensing Pharmacist Accreditation

Dispensing Pharmacist Signature

Physician Name

Physician Accreditation

Physician Signature

GWANGJU 2019

FINA

WORLD CHAMPIONSHOPS

Medical Center Drug Information Book

(EnglishVer.)

1. Central nervous system agents

1-1. Midazolam

Brand name: Midazolam<br>Major indications: hypnotics and sedatives<br>Available forms: 5mg/5ml/Ampoule<br>Dosage:-preoperative sedation) 0.07-0.08/kg, intramuscular injection 1 hour before surgery<br>-Endoscopy or cardiovascular treatments) initially 2-2.5 mg (0.035 mg/kg) for 2-3 minutes by intravenous injection<br>Route: intramuscular or intravenous injection<br>Side effects: laryngospasm, bronchospasm,bigeminy, retrospective amnesia, drowsiness,urticaria, etc.<br>Status: permitted

1-2. Dimenhydrinate

Brand name: Bonaling-A<br>Major indications: nausea, vomiting,dizziness<br>Available forms: 50mg/tablet<br>Dosage:50mg, 3-4 times a day, 50-100mg before 0.5-1 hour for the prevention of symptom<br>Route: oral<br>Side effects: drowsiness, headaches, dizziness, visual disturbance, rash, heartburn,thirst, etc.<br>Status: permitted

1-3. Zolpidem

Brand name: Stilnox<br>Major indications: hypnotics and sedatives<br>Available forms: 10mg/Tablet<br>Dosage:10mg<br>Route: oral<br>Side effects: drowsiness, headaches, dizziness, rebound insomnia etc.<br>Status: permitted

1-4. Lorazepam

Brand name: Ativan<br>Major indications: hypnotics and sedatives, tranquilizer (Anxiolytics)<br>Available forms: 4 mg/1 ml/Ampoule<br>Dosage:-Tab) 1–4mg daily in divided doses, increased if necessary to 10 mg<br>-Inj) preanesthetic medication: 0.05mg/kg by intravenous or intramuscular injection<br>anxiety and agitation: 0.025-0.03mg/kg, every 6 hours<br>Route: oral, intravenous or intramuscular injection<br>Side effects: dependency and withdrawal symptoms, drowsiness, dizziness, headaches, hypotension, etc.<br>Status: permitted

2. Analgesics and anti-inflammatory agents

2-1.Aceclofenac

Brand name: Airtal
Major indications: NSAIDs
Available forms: 100mg/Tablet
Dosage:1 tablet, 2 times daily
Route: oral
Side effects: digestive ulcers, indigestion, abdominal pain, nausea, dizziness, liver enzyme elevation, etc.
Status: permitted

2-2. Acetaminophen ER

Brand name: Tacenol ER
Major indications: mild to moderate pain, fever
Available forms: 650 mg/Tablet
Dosage: 2 tablets, 3 times daily
Route: oral
Side effects: nausea, hypersensitivity, anaphylaxis symptoms, rashes, blood disorders, liver damage, etc.
Status: permitted

2-3. Dexibuprofen

Brand name: Mexibupen ER tab
Major indications: NSAIDs
Available forms: 300 mg/Tablet
Dosage:2 tablets, 2 times daily (maximum of 1200 mg daily)
Route: oral
Side effects: indigestion, abdominal distractions, heartburn
Status: permitted

2-4.Diclofenac Sodium

Brand name: Toraren
Major indications: NSAIDs
Available forms: 75mg/2ml/Ampoule
Dosage: once daily (twice a day if necessary)
Route: intramuscular injection
Side effects: shock, peptic ulcer, gastrointestinal bleeding, vomiting, abdominal pain, diarrhea, rash, swelling, etc.
Status: permitted

2-5. Ibuprofen 200 mg/Arginine 185mg

Brand name: Carol-F
Major indications: rheumatoid arthritis, osteoarthritis, back pain, headache, dysmenorrhea, etc.
Available forms: tablet containing Ibuprofen 200mg, Arginine 185mg
Dosage:1-2 tablets every 4-6 hours with water (maximum of 6 tablets daily)
Route: oral
Side effects: nausea, hypersensitivity, anaphylaxis, rash, etc.
Status: permitted

2-6. Ketorolac tromethamine

Brand name: Keromin

Major indications: NSAIDs
Available forms: 30mg/1 ml/ampoule
Dosage: initially 10mg, then 10–30mg every 4–6 hours as required; maximum 90mg daily
Route: intramuscular or intravenous injection
Side effects: nausea,vomiting, indigestion, diarrhea, constipation, edema, itching, headache,drowsiness, dizziness, etc.
Status: permitted

2-7. Streptokinase .Streptodornase

Brand name: Mucolase
Major indications: anti inflammatory enzyme
Available forms: 10mg/Tablet
Dosage:1-2 tablets, 4 times daily
Route: oral
Side effects: hypersensitivity, rash, anaphylactic reaction, diarrhea, vomiting, blood disorder etc.
Status: permitted

2-8. Tramadol HCL

Brand name: Tramadol HCl Hanall
Major indications: moderate to severe pain (cancer), post-diagnostic and postoperative pain
Available forms: 50mg/1ml/Ampoule
Dosage:50~100mg, once daily (maximum 400mg daily)
Route: intramuscular or intravenous injection
Side effects: seizures, palpitation, shock, anaphylaxis, drowsiness, discomfort, nausea, vomiting, etc.
Status: permitted

2-9. Celecoxib

Brand name: Celebrex
Major indications: NSAIDs
Available forms: 200mg/Capsule
Dosage:200-400 mg daily
Route: oral
Side effects: dizziness, headache, indigestion, abdominal pain, nausea, rash, edema, insomnia, etc.
Status: permitted

2-10. Loxoprofen Sodium Hydrate

Brand name: Lenox Tab
Major indications: NSAIDs
Available forms: 60mg/T
Dosage:1 tablet, 3 times daily.
Route: oral
Side effects::hypersensitivity,anaphylactic reaction,peptic ulcer,headache,drowsiness, dizziness, etc.
Status: permitted

3. Skeletal muscle relaxants

3-1. EperisoneHCl

Brand name: Mulex<br>Major indications: muscle relaxants<br>Available forms: 50mg/Tablet<br>Dosage: 1 tablet, 3 times daily<br>Route: oral<br>Side effects: shock, rash, insomnia, drowsiness, headache, paralysis, nausea, vomiting, etc.<br>Status: permitted

3-2. Gallaminetriethiodide

Brand name: Gallamint<br>Major indications: muscle relaxants<br>Available forms: 10 mg/Ampoule<br>Dosage:10-20mg, repeated every 8-12 hours for 3 days<br>Route: intramuscular or intravenous injection<br>Side effects: Anaphylaxis, hypertension, tachycardia, dyspnea, etc.<br>Status: permitted

4. Autonomic nervous system agents

4-1. Cimetropium bromide

Brand name: Bropiuminj (5mg/ml), Alpium Tab (50mg/Tab)
Major indications: anticonvulsant
Available forms: 50mg/Tablet, 5mg/1ml/Ampoule
Dosage:-tab) 5mg, 3 times daily
-Inj) 5mg by intramuscular or intravenous injection
Route: oral, intramuscular or intravenous injection
Side effects: xerostomia, dysuria,headaches, dizziness, constipation, nausea, etc.
Status: permitted

4-2. Epinephrine

Brand name: Epinephrine
Major indications: bronchial asthma, serum sickness, urticaria, angioneurotic edema, cardiac arrest, lasting effect of local anesthetics
Available forms: 1mg/1ml/Ampoule
Dosage: adjusted according to the symptoms,
Side effects: palpitation, tachycardia, dyspnea, dizziness, anxiety, tremor, etc.
Status: prohibited (S6. stimulants)

4-3. Norepinephrine bitartrate

Brand name: Norpin
Major indications: acute hypotension, shock, cardiac arrest
Available forms: 10ml/Ampoule
Dosage: 8 mg (4 mg as NE) diluted to 5% in DW, 5% DS 1000ml, slow intravenous injection, at a rate of 2-3ml/minute, adjusted as necessary, followed by 0.5-1 ml/minute
Route: intravenous injection
Side effects: palpitation, bradycardia, headache, dizziness, anxiety, tremor, nausea, vomiting, etc.
Status: permitted

4-4. Atropine sulfate

Brand name: Atropine Sulfate
Major indications: anticonvulsant
Available forms: 0.5mg/1ml/Ampoule
Dosage: 0.5 mg by subcutaneous or intramuscular injection. intravenous injection if necessary.
Route: subcutaneous, intramuscular or intravenous injection
Side effects: dilation of pupils with loss of accommodation,palpitation, headache, dry mouth, nausea, vomiting, rashes, dysuria, etc.
Status: permitted

5. Cardiovascular agents

5-1. Amlodipine besylate

Brand name: Norvasc
Major indications: hypertension (Calcium channel blocker)
Available forms: 5mg/Tablet
Dosage:5mg, once daily, maximum 10 mg daily
Route: oral
Side effects: flush, fatigue, edema, dizziness, headache, stomachache, nausea, palpitation, etc.
Status: permitted

5-2. Aspirin

Brand name: Aspirin Protect Tab
Major indications: antiplatelet agent
Available forms: 100mg/T
Dosage:100 mg once daily,
Route: oral
Side effects: shock, hypersensitivity, peptic ulcer
Status: permitted

5-3. Atenolol

Brand name: Tenormin
Major indications: hypertension (beta blocker)
Available forms: 25mg/Tablet
Dosage:-hypertension) 50 mg, once daily, maximum 100 mg daily
-angina pectoris) 100mg daily in 1–2 divided doses, maximum 100 mg daily
Route: oral
Side effects: bradycardia, palpitation, rash, itching, dizziness, edema, etc.
Status: prohibited in particular sports (P2. Beta-blockers)

5-4. Captopril

Brand name: Capril
Major indications: hypertension (ACE inhibitor)
Available forms: 25mg/Tablet
Dosage:-hypertension) 50-100mgdaily in 1–2 divided doses, moderate to severe 100mg daily,
maximum 450 mg daily
-congestiveheart failure) 25-50mg, 3 times a day
Route: oral
Side effects: renal disorders, cytopenia, rash, dizziness, nausea, vomiting, liverdisorder, hyperkalemia, etc.
Status: permitted

5-5. Carvedilol

Brand name: Dilatrend
Major indications: hypertension (Alpha, Beta blocker)
Available forms: 12.5mg/Tablet
Dosage:- essential hypertension) 12.5-25mg, maximum single dose 25 mg, maximum 50 mg daily
- Chronic Stable Angina Pectoris) 12.5-50mg, twice daily
-congestiveheart failure) 3.125-25mg, twice daily, maximum 50-100 mg daily

Route: oral
Side effects: dizziness, headache, fatigue, bradycardia, hypotension, asthma, seizures, nausea, etc.
Status: prohibited (P2. Beta-blockers)

5-6. Diltiazem HCl

Brand name: Herben Retard Tab
Major indications: angina pectoris, hypertension (calcium channel blocker)
Available forms: 90mg/Tablet
Dosage:90mg, twice daily, maximum single dose 90-180mg
Route: oral
Side effects: headaches, rhinitis, sore throat, constipation, coughing, edema, diarrhea, myalgia, fatigue, indigestion, etc.
Status: permitted

5-7. Dopamine HCl

Brand name: Dopramine
Major indications: adrenergic agent
Available forms: 200mg/5ml/Ampoule
Dosage:2-5mg/kg/min by intravenous injection, increasing 5-10mg/kg/min or 20-50 mg/kg/min in severe case.
Route: intravenous injection
Side effects: arrhythmia, peripheral ischemia, palpitation, dyspnea, nausea,vomiting, abdominal pain, headache, anxiety, etc.
Status: permitted

5-8. Furosemide

Brand name: Furix, Lasix
Major indications: diuretics
Available forms: 40mg/Tablet, 20mg/2ml/Ampoule
Dosage:-Tab) 20-80mg daily, maximum 600 mg daily in severe case, maximum 80 mg in hypertension
-Inj) 20-40mg, IV or IM daily
Route: oral, intramuscular or intravenous injection
Side effects: orthostatic hypotension, dizziness, headache, anorexia, rash, fatigue, etc.
Status: prohibited (S5. diuretics and masking agents)

5-9. Losartan potassium

Brand name: Sarlotan Tab 50mg
Major indications: hypertension (Angiotension ll Receptor Antagonists)
Available forms: 50mg/Tablet
Dosage:50 mg, once daily, maximum single dose 100mg daily
Route: oral
Side effects: muscle pain, dizziness, headache, upper respiratory tract infection, indigestion, diarrhea, cough, fatigue, etc.
Status: permitted

5-10. Nicorandil

Brand name: Sigmart
Major indications: angina pectoris
Available forms:5mg/Tablet
Dosage:5mg, 3 times daily
Route: oral
Side effects: headache, nausea, vomiting, dizziness, rash, fatigue, etc.
Status: permitted

5-11. Nifedipine

Brand name: AdapineOsmo SR 30mg
Major indications: angina pectoris, hypertension (Calcium channel blocker)
Available forms: 30mg/Tablet
Dosage:30-60mg daily, maximum 120mg daily
Route: oral
Side effects: headaches, dizziness, chest pains, palpitation, flushing, indigestion, edema, paresthesia, etc.
Status: permitted

5-12. Nitroglycerin

Brand name: Nitroglycerin Sublingual tab, Nitrolingual inj
Major indications: treatment or prevention of angina pectoris
Available forms: 0.6mg/Tablet, 50mg/50ml/IV
Dosage:-Tab) 0.3mg-0.6mg by sublingual
(repeat every 5 minutes if needed for a total of 3 tablets in 15 minutes)
-Inj) 10-20mg/min, if necessary, to maximum 400mg/min
Route: Sublingual, intravenous injection
Side effects: hypotension, palpitation, rash, headache, nausea, vomiting, etc.
Status: permitted

5-13. Valsartan

Brand name: Diovan
Major indications: hypertension (Angiotensin Ⅱ Receptor Antagonists)
Available forms: 80mg/Tablet
Dosage:-essential hypertension) 80mg, once daily, maximum 160-320mg daily
-heart failure) 40-160mg, twice daily, maximum 320mg daily
Route: oral
Side effects: BUN elevation, headaches, dizziness, coughing, indigestion, diarrhea, fatigue,myalgia, etc.
Status: permitted

5-14. Amiodarone HCl

Brand name: Cordarone
Major indications: ventricular arrhythmias
Available forms: 150mg/3ml/Ampoule
Dosage:-Tab) initially 600mg once daily for 8-10 days, Increased up to800-1000mg if necessary
-Inj) 5-10 mg/kg + dextrose injection 5% 250ml
Route: oral, intravenous injection
Side effects: bradycardia, hypotension, etc.
Status: permitted

5-15. Spironolactone

Brand name: Aldacton
Major indications: diuretics
Available forms: 25mg/Tablet
Dosage: 50-100mg daily in divided doses
Route: oral
Side effects: nausea, vomiting, myospasm, gynecomastia, skin rash, dizziness, fatigue, diarrhea, etc.
Status: prohibited (S5. diuretics and masking agents)

6. Endocrine system agents

6-1. Glimepiride

Brand name: Euglex
Major indications: diabetes mellitus(Sulfonylureaclass)
Available forms: 2mg/Tablet
Dosage:initially1mg, once daily, maintenance 1-4 mg daily, maximum 8 mg daily
Route: oral
Side effects: hypoglycemia, visual disturbances,abdominal pain,nausea, vomiting, allergy, dizziness, etc.
Status: permitted

6-2. Insulin

Brand name: Humulin 70/30 PEN, Humalog Vial
Major indications: diabetes mellitus
Available forms: Insulin human(RI30:NPH70): Humulin70/30 pen 300 IU/3 ml
Insulin lispro: Humalog 1000IU/10ml
Dosage: according to requirements
Side effects: hypoglycemia, hypersensitivity, neuralgia, injection site reaction,eye refractive error,etc.
Status: prohibited (S4. Hormone and metabolic modulators)

6-3. Metformin HCl

Brand name: Diabex XR
Major indications: diabetes mellitus (Biguanide class)
Available forms: 500mg/Tablet
Dosage:500mg, 2-3 times daily, maximum 2,000 mg daily
Route: oral
Side effects: lactic acidosis, diarrhea, abdominal pain, nausea, vomiting, dysgeusia, hypoglycemia, etc.
Status: permitted

7. Hormones and antagonistic agents

7-1. Prednisolone

Brand name: Solondo
Major indications: adrenal cortical hormone
Available forms: 5mg/Tablet
Dosage:5-50 mg daily in 1-4 divided doses
Route: oral
Side effects: peptic ulcer, heartburn, insomnia, acne, edema, diabetes, etc.
Status: prohibited (S9. Gluticocorticoids)

7-2. Methylprednisolone Sodium succinate

Brand name: Solumedrol, Predisol
Major indications: adrenal cortical hormone
Available forms: 125 mg/Vial, 500mg/Vial
Dosage: adjusted according to response
Route: intravenous injection
Side effects: gastric ulcer, edema, hyperglycemia, rash, acne, cushing syndrome, convulsions, etc.
Status: prohibited (S9. Gluticocorticoids)

7-3. Dexamethasone Sodium phosphate

Brand name: Dexamethasone
Major indications: adrenal cortical hormone
Available forms: 5mg/1ml/Ampoule
Dosage:IV,IM) 2-8mg, every 3-6 hours
infusion)2-10 mg, once or twice daily
Route: intravenous or intramuscular injection
Side effects: peptic ulcer, heartburn, insomnia, acne, edema, diabetes, etc.
Status: prohibited

8. Respiratory system agents

8-1. Acetylcysteine

Brand name: Moktin cap, Spatam
Major indications: Antitussive and mucoactive drugs
Available forms: 200mg/capsule, 300mg/3ml/ampoule
Dosage:-Capsule) 200mg, 2-3 times daily
-Injection)IV: 600-900mg, 2-3 times daily
(preparation: diluted with an equal volume of 0.9% normal saline or dextrose solution)
IM: 300mg, once or twice daily
Route: oral, intravenous or intramuscular injection
Side effects: nausea, vomiting, diarrhea, hypersensitivity reaction, etc.
Status: permitted

8-2. Ambroxol HCl

Brand name: Ambrect, Aroxol
Major indications: Antitussive and mucoactive drugs
Available forms: 30mg/Tablet, 15mg/2ml/Ampoule
Dosage:-Tablet) 30mg, 2-3 times a day
-Injection) 15-30 mg, 2-3 times by subcutaneous, intramuscular or slow intravenous injection
Route: oral, intramuscular or intravenous injection
Side effects: damaged skin, indigestion, nausea, vomiting, dry mouth, dysgeusia, etc.
Status: permitted

8-3. Budesonide + FormoterolFumarateDihydrate

Brand name: Symbicort Turbuhaler 160/4.5mg
Major indications: asthma, maintenance and reliever therapy
Available forms: (Budesonide 160mg, Formoterol 4.5mg) per time, 120 does/bottle
Dosage:1–2 puffs once or twice daily, increased if necessary to maximum 4 puffs/time, 8 puffs/day
Route: inhalation
Side effects: headache, tremor, palpitation, nasopharyngeal stimulation, hoarseness, oral candidiasis, etc.
Status: prohibited

8-4. Fluticasone propionate

Brand name: Flixotide Diskus
Major indications: asthma (steroidal drugs)
Available forms: 250mg/metered inhalation, 60 does/Bottle
Dosage:100-1000mg, twice daily
Route: inhalation
Side effects: oral candidiasis, hoarseness, hypersensitive reaction of skin, etc.
Status: permitted

8-5. Salbutamol sulfate

Brand name: Ventolin Evohaler
Major indications: asthma (bronchial dilator)
Available forms: 100mg/metered inhalation, 200 dose/bottle
Dosage: by aerosol inhalation: 100microgram (1 puffs), 2 puffs if necessary. For persistent symptoms, up to 2 puffs/time, maximum 4 times daily,
Route: inhalation
Side effects: headache, tremor, tachycardia, nasopharyngeal stimulation, hypersensitivity, myalgia, etc.

Status: prohibited (S3. Beta-3 agonist)

8-6. Erdosteine

Brand name: Erdos
Major indications: Antitussive and mucoactive drugs
Available forms: 300mg/Capsule
Dosage: 1 capsule, 2-3 times daily.
Route: oral
Side effects: fatigue, indigestion, stomachache, gastro-intestinal disturbances, diarrhea, vomiting, etc.
Status: permitted

8-7. Caffeine anhydrous + Chlorpheniramine maleate + Dihydrocodeine Tartrate + Dl-methylephedrine HCl

Brand name: Neo-Medicough
Major indications: cough, sputum,
Available forms: Caffeine anhydrous 10mg, Chlorpheniramine maleate 1.5mg, Dihydrocodeine Tartrate 5mg, and Dl-methylephedrine HCl 17.5 mg/tablet
Dosage: 2 tablets, 3 times daily after meals.
Route: oral
Side effects: rash, redness, nausea, vomiting, constipation, drowsiness, anorexia, etc.
Status: prohibited (S6. Stimulants)

8-8. Theobromine

Brand name: Anycough
Major indications: rhinitis, sinusitis, cough by acute/chronic bronchitits
Available forms: 300mg/Capsule
Dosage:1 capsule, twice daily
Route: oral
Side effects: itching, rash, drowsiness, abdominal distension, hyperuricemia, edema, etc.
Status: permitted

9. Antihistamines

9-1. Fexofenadine HCl

Brand name: Allegra
Major indications: Antihistamines(120 mg: seasonal allergic rhinitis; 180 mg: urticarial)
Available forms: 180mg/Tablet
Dosage:1 tablet, once daily
Route: oral
Side effects: headaches, drowsiness, back pain, sleep disorders, rashes, urticaria, fatigue, etc.
Status: permitted

9-2.Levocetirizine HCl

Brand name: Xyzal
Major indications: Antihistamines
Available forms: 5mg/Tablet
Dosage:5 mg once daily
Route: oral
Side effects: drowsiness, fatigue, headaches, dizziness,dry mouth, nausea, indigestion, abdominal pain, palpitation, etc.

Status: permitted

9-3. Chlorpheniramine Maleate

Brand name: Peniramin, Chlorpheniramine Maleate Inj<br>Major indications: Antihistamines<br>Available forms: 2mg/Tablet, 2mg/2ml/Ampoule<br>Dosage: Tab) 2-6 mg, 2-4 times a day<br>Inj) 5-10mg, 1-2 times a day via SC, IM or IV<br>Route: oral, subcutaneous, intramuscular or intravenous injection<br>Side effects: cyanosis, shock, convulsions, confusion, rashes, oliguria, hemolytic anemia, nausea, vomiting, etc.<br>Status: permitted

10. Dental agents

10-1. Benzydamine HCl

Brand name: Tantum verde nebulizer
Major indications: painful inflammatory conditions of oropharynx
Available forms: 1.5ml/ml, 30ml/bottle
Dosage: 2-6 times daily,
Route: oral rinse or gargle
Side effects: occasional numbness or stinging, rarely hypersensitivity reactions
Status: permitted

10-2. Chlorhexidinegluconate

Brand name: Hexamedin
Major indications: gingivitis, stomatitis
Available forms: 1mg/g, 250ml/bottle
Dosage: 15 ml, twice daily, toothbrushing for 1 minutes
Route: oral rinse or gargle
Side effects: rash, staining of teeth and mucous membranes, etc.
Status: permitted

11. Gastro-intestinal system agents

11-1. Diomagnite

Brand name: Diogel Suspension
Major indications: Antacids
Available forms: 3.8g/17g/pack
Dosage: 1 pack after meals or when symptoms occur, dissolved in half a cup of water
Route: oral
Side effects: discomfort, phosphorous metabolism disorder, diarrhea, etc.
Status: permitted

11-2. Bacillus Subtillis + Streptococcus faecium

Brand name: Medilac DS
Major indications: probiotics
Available forms: 250mg/enteric capsule
Dosage: 1 capsule, 2-3 times daily
Route: oral
Status: permitted

11-3. Bromelin + Dimethicone + Pancreatin

Brand name: Beszyme
Major indications: digestant
Available forms: 1 tablet (Bromelin 30mg, Dimethicone 40mg, Pancreatin 400mg)
Dosage: 1 tablet, 3 times daily
Route: oral
Status: permitted

11-4. Bisacodyl + Docusate sodium

Brand name: Duorax
Major indications: Laxatives
Available forms: 1 tablet (Bisacodyl 5mg, Docusate sodium 16.75mg)
Dosage: 2 tablets daily
Route: oral
Side effects: stomachache, diarrhea, vomiting, etc.
Status: permitted

11-5. Dioctahedralsmectite

Brand name: Smecta Suspension
Major indications: Acute diarrhea
Available forms: 3 g/20 ml/Pack
Dosage: 20 ml, 3 times daily
Route: oral
Side effects: constipation
Status: permitted

11-6. Loperamide HCl

Brand name: Loperamide HCl
Major indications: Acute diarrhoea
Available forms: 2mg/Capsule
Dosage: initially 4 mg, followed by a further 2 mg if necessary, usual dose 2–8mg daily, maximum 16

mg daily
Route: oral
Side effects: constipation, dizziness, nausea, abdominal cramps, ect.
Status: permitted

11-7. Magnesium hydroxide

Brand name: Magmil
Major indications: Antacids, laxatives
Available forms: 500mg/Tablet
Dosage:- constipation) 2-4 tablets daily, 1-2 divided doses
-dyspepsia) 2-5 tablets daily, # divided doses
Route: oral
Side effects: diarrhea, etc.
Status: permitted

11-8. Metoclopramide HCl

Brand name: Mecool
Major indications: gastroesophageal reflux
Available forms: 10mg/2ml/Ampoule
Dosage: 10 mg by intramuscular injection or by intravenous injection over at least 3 minutes, maximum 0.5 mg/kg or 30 mg daily
Route: intramuscular or intravenous injection
Side effects: stomachache, diarrhea, constipation, fatigue, headache, shock, malignant neuroleptic syndrome, etc.
Status: permitted

11-9. Mosapridecitrate

Brand name: Gasmotin
Major indications: gastroesophageal reflux
Available forms: 5mg/Tablet
Dosage:15 mg daily, 3 divided doses
Route: oral
Side effects: diarrhea, dry mouth, malaise, liver function disorder, hypersensitivity, etc.
Status: permitted

11-10. Ranitidine HCl

Brand name: Curan
Major indications: peptic ulcer (H2 blocker)
Available forms: 150mg/Tablet
Dosage: 1 tablet daily, 2 times a day
Route: oral
Side effects: constipation, diarrhea, itching, eosinophilia, malaise, fever, etc.
Status: permitted

11-11. Sucralfate

Brand name: Ulcerlmin suspension
Major indications: : peptic ulcer
Available forms: 1g/15ml/Pack
Dosage: 1 pack, 3-4 times daily
Route: oral

Side effects: constipation, dry mouth, nausea, etc.
Status: permitted

11-12. Trimebutine maleate

Brand name: Spabutin
Major indications: gastroesophageal reflux, antispasmodics
Available forms: 100mg/Tablet
Dosage: 1-2 tablets, 3 times daily before meals
Route: oral
Side effects: fatigue, diarrhea, nausea, vomiting, constipation, etc.
Status: permitted

12. Antimicrobial agents

12-1. Cefazoline sodium

Brand name: Cefazolin
Major indications: 1st generation of cephalosporins
Available forms: 1g/Vial
Dosage: 1g daily in 2 divided doses, maximum 5g daily
Route: intravenous or intramuscular injection
Side effects: nausea, vomiting, rash, urticarial,anaphylaxis etc.
Status: permitted

12-2. Cefroxadine

Brand name: Tiroxin
Major indications: 1st generation of cephalosporins
Available forms: 250mg/Capsule
Dosage:250-500 mg, 2-3 times daily
Route: oral
Side effects: nausea, vomiting, diarrhea, rashes, urticarial, increased liver enzymes etc.
Status: permitted

12-3. Cefaclor

Brand name: Cefaclor
Major indications: 2nd generation of cephalosporins
Available forms: 250mg/Capsule
Dosage: 250 mg every 8 hours
Route: oral
Side effects: anaphylaxis (systemic lupus erythematosus, edema, dyspnea, angioedema), rash, urticarial, Stevens-Johnson syndrome, nausea, vomiting, diarrhea, etc.
Status: permitted

12-4. Ceftriaxone sodium

Brand name: Ceftriaxone
Major indications: third generation of cephalosporins
Available forms: 1g/Vial, 2g/Vial
Dosage:1-2g daily by intravenous or intramuscular injection, maximum 4 g daily
Route: intravenous or intramuscular injection
Side effects: nausea, vomiting, diarrhea, dizziness, increased liver enzymes, rash, etc.
Status: permitted

12-5. CefditorenPivoxil

Brand name: Meiact
Major indications: third generation of cephalosporins
Available forms: 100mg/Tablet
Dosage: 100 mg, 3 times daily
Route: oral
Side effects: nausea, vomiting, diarrhea, rash, urticarial, eosinophilia, etc.
Status: permitted

12-6. Metronidazole

Brand name: Furacinil
Major indications: antiprotozoal agent

Available forms: 250mg/Tablet
Dosage:-trichomoniasis) 250mg, 2 times daily for 10 days
-amoebiasis) 750mg, 3 times daily for 5-10 days
-treatment of anaerobic bacteria) 500mg, 3-4 times a day for 7-10 days, maximum 4g daily.
Route: oral
Side effects: rash, itching,headache, convulsions,confusion, visual disturbance, ect.
Status: permitted

12-7. Acyclovir

Brand name: Aclova
Major indications: Antiviral drugs
Available forms: 400mg/Tablet
Dosage:200-800 mg,2-6 times daily
Route: oral
Side effects: hypersensitivity, rash, fever, nausea, vomiting, diarrhea, etc.
Status: permitted

12-8. Amoxicillin sodium + Clavulanate potassium

Brand name: Augmentin(375mg), Moxicle(625mg),
Major indications: penicillinsantibacterial drugs
Available forms: 375 mg/tablet (Amoxicillin 250 mg, Clavulanate potassium 125 mg)
625mg/ tablet (Amoxicillin 500mg, Clavulanate potassium 125mg)
1.2g/vial (Amoxicillin 1g, Clavulanate potassium 200mg)
Dosage:Tab) 250-500 mg as a Amoxicillin, three times daily
Inj) 1.2g every 8 hours, every 6 hours in more severe infections
Route: oral, intravenous injection
Side effects: diarrhea, pseudomembranous colitis, stomatitis, indigestion, rash, dermatitis, etc.
Status: permitted

12-9. Ciprofloxacin

Brand name: Cycin
Major indications: Quinolones antibacterial drugs
Available forms: 250mg/Tablet
Dosage:250-500mg, twice daily, increased in severe multiple infections to 750mg, twice daily Route: oral
Side effects: diarrhea, nausea, vomiting, headache, dizziness, restlessness, hypersensitivity, etc.
Status: permitted

12-10. Ciprofloxacin

Brand name: Cycin
Major indications: Quinolones antibacterial drugs
Available forms: 400mg/200ml/Bag
Dosage: 100-400 mg, twice daily, by intravenous injection over at least 60 minutes.
Route: intravenous injection
Side effects: diarrhea, nausea, vomiting, abdominal ache, headache, dizziness, restlessness, hypersensitivity, etc.
Status: permitted

12-11. Levofloxacin

Brand name: Cravit

Major indications: : Quinolones antibacterial drugs
Available forms: 100mg/Tablet
Dosage:100mg, 2-3 times a day, increased up to200mg every 8 hours if necessary.
Route: oral
Side effects: hypersensitivity, insomnia, dizziness, headache, nausea, vomiting, diarrhea, abdominal ache, etc.
Status: permitted

12-12. Doxycycline monohydrate

Brand name: Doxycycline
Major indications: tetracyclines antibacterial drugs
Available forms: 100mg/Tablet
Dosage: initially 100 mg every 12 hours, followed by 100 mg daily 1–2 divided doses
Route: oral
Side effects: anemia, anorexia, hypersensitivity, nausea, vomiting, diarrhea, etc.
Status: permitted

13. Ophthalmic agents

13-1. Flouorometholone

Brand name: Ocumetholone eye drop
Major indications: eye drops (corticosteroids)
Available forms: 1mg/1ml, 5ml/bottle
Dosage: apply one or two drops, 2-4 times daily
Route: ocular
Side effects: raised intra-ocular pressure, red eye, irritation, etc.
Status: permitted

13-2. Levofloxacin

Brand name: Cravit eye drop 1.5% 5mL
Major indications: eye drops (bacterial conjunctivitis)
Available forms: 5mg/ml, 5ml/bottle
Dosage: apply one drop, 3 times daily
Route: ocular
Side effects: irritation, itching, red eye, etc.
Status: permitted

13-3. Ofloxacin

Brand name: Quinovid EYE OINT 5g
Major indications: eye ointment (bacterial conjunctivitis)
Available forms: 3.5g/TU(3mg/1g)
Dosage: apply one drop, 3 times daily
Route: ocular
Side effects: irritation, itching, red eye, etc.
Status: permitted

13-4. Olopatadine HCl

Brand name: Olotadine Eye Drop
Major indications: eye drops(allergic conjunctivitis)
Available forms: 1.11mg/ml, 5ml/bottle
Dosage: apply one drop, twice daily
Route: ocular
Side effects: cough, pharyngitis,blurred vision,abnormal sensation in eyes, etc.
Status: permitted

13-5. Sodium hyaluronate

Brand name: Hyaludrop Eye Drop
Major indications: artificial tear substitute
Available forms: 1mg/ml, 10ml/BT, 0.5ml/1tube
Dosage: apply one drop, 5-6 times daily
Route: ocular
Side effects: irritation, itching eyelid, bleeding, eye infection, etc.
Status: permitted

13-6. Tropicamide + Phenylephrine HCl

Brand name: Mydrin-P OphSoln
Major indications: Mydriatics
Available forms: 10ml/bottle, Tropicamide 5 mg and phenylephrine HCl 5mg in 1ml

Dosage:Pupil Dilation)apply one or two drops one time or one drop every 3-5 minutes 2-3 times.
Cycloplegia)apply one drop every 3-5 minutes 2-3 times.
Route: ocular
Side effects: conjunctivitis,raised intra-ocular pressure, itching, rash, urticaria, etc.
Status: permitted

13-7. Neomycin Sulfate + Polymixin B Sulfate + Dexamethasone

Brand name: Forus eye oint 5g
Major indications: eye drops (antimicrobial agent with steroids)
Available forms: 5ml/bottle, Dexamethasone 1 mg, Neomycin sulfate 3.5 mg(3500 IU) and Polymyxin B sulfate6,000 IU in 1ml
Dosage:apply one or two drops, 4-6 times daily
Route: ocular
Side effects: allergic reaction, raised intra-ocular pressure, etc.
Status: prohibited

13-8. Proparacaine HCl 5mg

Brand name: Alcain eye drop
Major indications: topical anesthesia for tonometry
Available forms: 5mg/ml, 15ml/bottle
Dosage: Intraocular pressure measurement and removal of foreign substance and suture)apply 1-2 times before surgery.
Deep sedation for ophthalmic surgery)apply one drop every 5-10 minute, 5-7 times
Route: ocular
Side effects: irritation, burning sensation of eyes, eye redness, etc.
Status: permitted

13-9. Moxifloxacin HCl

Brand name: Vigamox eye drop
Major indications: eye drops (bacterial conjunctivitis)
Available forms: 5.45mg/ml, 5ml/bottle
Dosage: apply one drop, 3-5 times daily
Route: ocular
Side effects: conjunctivitis, eye pain, fever, cough, infection, shock, anaphylactic reaction, etc.
Status: permitted

14. Otorhinolaryngologicalagents

14-1. Azelastine HCl

Brand name: Azeptin nasal spray
Major indications: allergic rhinitis (antihistamines)
Available forms: 0.14 mg/metered spray, 10 mg/bottle
Dosage:1 spray into each nostril twice daily
Route: intranasal
Side effects: taste disturbances, headache, nasalburning sensation, epistaxis, etc.
Status: permitted

14-2. Fluticasone furoate

Brand name: Avamys nasal spray
Major indications: allergic rhinitis (steroids)
Available forms: 27.5mg/ metered spray, 120 spray/bottle
Dosage:-initially)2 sprays into each nostril once daily
-maintenance)1 spray into each nostril once daily
Route: intranasal
Side effects: headache, epistaxis, nasopharyngitis, etc.
Status: permitted

14-3. Ofloxacin

Brand name: Effecxinoticsoln
Major indications: ear drops(otitis media, otitis externa)
Available forms: 3mg/ml, 5ml/bottle
Dosage:6-10 drops into the affected ear,twice daily, lie down with the affected ear uppermost for 10 minutes.
Route:intraear
Side effects: ear pain, local sensitivity reactions, etc.
Status: permitted

15. Dermatological agents

15-1. Acyclovir

Brand name: Vivir cream
Major indications: antivirals (herpes simplex and varicella-zoster)
Available forms: 50mg/g, 5g/tube
Dosage: every 4 hours, apply 5 times daily for 5 days
Side effects: contact dermatitis, rash, eczema, allergic reaction, etc.
Status: permitted

15-2. Sertaconazole Nitrate

Brand name: Dermofix Cream
Major indications: external antifungal agent (fungal skin infections)
Available forms: 20mg/g, 30g/tube
Dosage: apply once or twice daily
Side effects: redness, burning, itching, etc.
Status: permitted

15-3. Mupirocin

Brand name: Bearobanoint
Major indications: external antibacterial drugs (skin infection)
Available forms: 20mg/g, 10g/Tube
Dosage: apply smaller amounts, 2-3 times daily
Side effects: local irritation, itching, rashes, fever, dry skin, etc.
Status: permitted

15-4. Prednicarbate

Brand name: Titibeoint
Major indications: external steroids (steroid responsive dermatoses)
Available forms: 2.5mg/g, 10g/Tube
Dosage: once or twice daily
Side effects: skin irritation, fever, itching, rashes, etc.
Status: permitted

15-5. Oxytetracycline HCl + Polymixin B sulfate

Brand name: Terramycinophoint
Major indications: eye ointment (antibacterial drugs)
Available forms: 3.5g containing Oxytetracycline hydrochloride 17.5mg, Polumyxin B sulfate 35000IU
Dosage: approximately 1.3 cm 2-4 times daily
Side effects: blepharitis,conjunctivitis, fever, etc
Status: permitted

15-6. Urea

Brand name: Urea Cream
Major indications: kerato dermiatylodes palmaris progressive,
Available forms: 200mg/g, 50g/Tube
Dosage: once to several times daily
Side effects: pain, heat, redness, hypersensitivity, eczema, skin cracks, papule, etc.
Status: permitted

15-7. Ketoprofen gel

Brand name: Rheuma gel, Kefentec-L Plasta
Major indications: external NSAIDs (ankylosing spondylitis, migraine, arthritis, pain, dysmenorrhea)
Available forms: 50g/Tube (30mg/g), 30mg/10cm*7cm 1sheet
Dosage:Gel) apply to lesions 1-4 times
Plasta) apply to lesions once
Side effects: hypersensitivity, rash, redness, itching, irritation, etc.
Status: permitted

15-8. Calamine + zinc oxide

Brand name: Calamin lotion
Major indications: relief and improvement of miliaria, erosion
Available forms: 100ml/bottle, 1ml containing Calamine 80mg and zinc oxide 80mg
Dosage: once to several times daily
Side effects: hypersensitivity, rash, redness, itching, irritation, etc.
Status: permitted

15-9. Beta sitosterol

Brand name: Mebooint
Major indications: burns, wounds
Available forms: 5mg/g, 40g/tube
Dosage: apply thinly to lesions 2-3 times daily
Side effects: pruritus, redness, swelling, papule, vesicle, etc.
Status: permitted

16. Vitamins

16-1. Vitamin (multi)

Brand name: M.V.H<br>Major indications: multi vitamin<br>Available forms: 5ml/Vial(Ascorbic 500 g, Dexpanthenol 25 g, Ergocalciferol 25 μg,<br>Nicotinamide 100mg, Pyridoxine HCl 15mg, Retinol Palmitate 6.65mg,<br>Riboflavin sodium phosphate 12.7mg, Thiamine HCl 50mg, Tocopherol acetate 5mg in 1 vial)<br>Dosage: by continuous intravenous infusion once daily, 5 ml in 500-1000 ml DW, NS for intravenous injection<br>Route: intravenous injection<br>Side effects: rash, nausea, vomiting, unconsciousness, fever, etc<br>Status: permitted

17. Emergency contraceptive agents

17-1. Levonorgestrel

Brand name: Norlevo one<br>Major indications: emergency contraceptives<br>Available forms: 1.5mg/Tablet<br>Dosage:1.5mg once<br>Route: oral<br>Side effects: dizziness, headache, nausea, weak abdominal pain, etc.<br>Status: permitted

18. Biological agents

18-1. Human anti-tetanus immunoglobulin

Brand name: Hypertet
Major indications: tetanus
Available forms: 250 IU/1ml/Vial
Dosage:prevention) 250IU once, relief of symptoms) 5000 IU
Route: intramuscular injection
Side effects: dyspnea, fever, rash, pain, hard labor, etc.
Status: permitted

18-2. Adsorbed Diphtheria Toxoid + Absorbed tetanus toxoid(TD)

Brand name: Dite Booster SSI
Major indications: prevention of diphtheria and tetanus
Available forms: 0.5ml/PFS
Dosage:- prevention of tetanus, after immunization against tetanus
After 5 to 10 years of immunization: 0.5ml,
After more than 10 years: 0.5ml + tetanus immunoglobulin 250 IU
Route: intramuscular injection
Side effects: redness, pain, cold symptom, rash, etc.
Status: permitted

19. Local anaesthetics

19-1. Lidocaine HCl

Brand name: Lidocaine
Major indications: local anesthetic
Available forms: 400mg/20ml/Vial
Dosage: depends on whether loading is required.
Route: intravenous injection
Side effects: shock, malignant hyperthermia, convulsions, drowsiness, dizziness, rash, etc.
Status: permitted

19-2. Lidocaine HCl + Epinephrine

Brand name: Lidocaine. Epinephrine (1:100,000)
Major indications: local anesthetic
Available forms: 1.8ml/Ampoule (Lidocaine HCl 36 mg + Epinephrine 18 mg in 1 ampoule)
Dosage:-nerve block or infiltration) 0.3-2ml
-oral surgery) 3-5ml
Route: intravenous injection
Side effects: shock, malignant hyperthermia, convulsions, drowsiness, dizziness, rash, etc.
Status: Epinephrine prohibited (S6. Stimulants)

19-3. Lidocaine

Brand name: Beracaine spray
Major indications: local anesthetic
Available forms: 10mg/1 metered spray, 10%50ml/bottle
Dosage:-adult) maximum 200mg (20 sprays)
-dentistry) 10~15 mg(1~5 sprays)
-otorhinolaryngology) 30mg (3 sprays)
-Insertion of catheters into respiratory tract or digestive organ)maximum 200mg (20 sprays)
-obstetrics and gynecology)maximum 200mg (20 sprays)
Route:aerosol spray
Side effects: shock, convulsions, drowsiness, dizziness, bradycardia, rash, etc.
Status: permitted

20. Fluids

20-1. Calcium chloride+ Potassium chloride + Sodium chloride + Sodium lactate

Brand name: Hartman soln
Major indications: Electrolyte solution Available forms: 500ml/BAG (Calcium chloride 135mg, Potassium chloride 200mg,
Sodium chloride 3g, Sodium lactate 1.6g in 500 ml solution)
Dosage:500-1,000 ml, intravenous infusion 300-500ml/hour,
Route: intravenous injection
Side effects: cerebral edema, pulmonary edema, peripheral edema, etc. in case of massive and rapid injection
Status: permitted

20-2. Dextrose

Brand name: Dextrose injection
Major indications: source of calories, sodium chloride, and water
Available forms: 10% 500ml(bag), 5% 1000ml(bag). 5% 500ml(bag)
Side effects: electrolyte imbalance, congestion, etc.
Status: permitted

20-3. Sodium chloride

Brand name: Normal saline
Major indications: Electrolyte solution
Available forms: (0.9%) 1000ml/bag, 500ml/bag, 100ml/PP
Side effects: congestive heart failure, edema, electrolyte imbalance, etc. in case of massive and rapid injection
Status: permitted

20-4. Sodium chloride

Brand name: Sodium chloride
Major indications: electrolytic correction of electrolyte supplement (it should be diluted before use)
Available forms: 2.34 g/20 ml/Ample(40 mEq)
Side effects: hypernatremia, congestive heart failure, edema, etc. in case of massive and rapid injection
Status: permitted

20-5. Water for injection

Brand name: Water for injection
Major indications: dilution or dissolving of drugs
Available forms: 20ml/Ampoule
Status: permitted

20-6. Sodium bicarbonate

Brand name: Sodium bicarbonate 8.4%
Major indications: acidosis, acute urticaria
Available forms: 1.68g/20ml/Ampoule
Side effects: alkalosis, dysesthesia, bradycardia, etc.
Status: permitted

21. Disinfectants

21-1. Providone Iodide

Brand name: Povidone 10%
Major indications: disinfection, sterilization
Available forms: 1000ml/Bottle
Status: permitted

21-2. Chlorhexidinegluconate 4%

Brand name: Microshield
Major indications: gingivitis, stomatitis
Available forms: 1.5L/each
Status: permitted

21-3.Povidone-iodine 0.15g/1.5 ㎖

Brand name: Povidone Stick Swab
Major indications: disinfection, sterilization
Available forms: 2EA/PKG
Status: permitted

21-4.Chlorhexidinegluconate solution/Ethanol

Brand name: Green HexidineStickswabsoln 2%
Major indications: disinfection, sterilization
Available forms: 2EA/PKG
Status: permitted
